# Hydrogen reverses the clustering tendency of carbon in amorphous silicon oxycarbide

**DOI:** 10.1038/srep13051

**Published:** 2015-08-13

**Authors:** Hepeng Ding, Michael J. Demkowicz

**Affiliations:** 1 Department of Materials Science and Engineering, Massachusetts Institute of Technology, Cambridge, MA 02139; 2 Institute of Fundamental and Frontier Sciences, University of Electronic Science and Technology of China, Chengdu 610054, China

## Abstract

Amorphous silicon oxycarbide (SiOC) is of great technological interest. However, its atomic-level structure is not well understood. Using density functional theory calculations, we show that the clustering tendency of C atoms in SiOC is extremely sensitive to hydrogen (H): without H, the C-C interaction is attractive, leading to enrichment of aggregated SiC_4_ tetrahedral units; with hydrogen, the C-C interaction is repulsive, leading to enrichment of randomly distributed SiCO_3_ tetrahedral units. Our results suggest that conflicting experimental characterizations of C distributions may be due to differing amounts of H present in the samples investigated. Our work also opens a path for tailoring the properties of SiOC by using the total H content to control the C distribution.

There are no known, thermodynamically stable, ternary compounds of silicon (Si), oxygen (O), and carbon (C)^1^. However, metastable amorphous silicon oxycarbide (SiOC) is readily synthesized through a variety of techniques^2^,^3^,^4^,^5^,^6^,^7^. This material is of great technological interest due to an unusual combination of properties^8^,^9^, including excellent thermal stability^10^ (T_crystallization_ ≈ 1500 K) and ease of processing in bulk quantities^11^. Potential applications include lithium ion batteries^12^, biotechnology^13^, nuclear energy^14^,^15^, and others^8^,^16^,^17^,^18^. One attractive attribute of SiOC is that it may be possible to systematically tune its properties, e.g. by adjusting the O/C ratio or by introducing dopants. However, the atomic-level structure of this material remains poorly understood, hindering efforts to control its behavior.

We investigate the distribution of C in a Si-O-C continuous random network using first principles density functional theory (DFT) calculations and show that it is highly sensitive to hydrogen (H): without H, C atoms in SiOC bind to each other and therefore tend to aggregate; with H, they repel and distribute uniformly throughout the network. Our finding suggests that H is the key to resolving discrepancies in experimental investigations of SiOC^19^,^20^,^21^. It also indicates that the distribution of C in SiOC may be tailored by controlling the amount of H in the material.

SiOC is a heterogeneous material composed of a Si-O-C continuous random network (CRN)^1^,^22^, SiC precipitates^19^, and C^19^ in the form graphite-like nano-domains^23^, graphene-like sheets^23^, or turbostratic C (a variant of hexagonal graphite)^24^. We focus specifically on the Si-O-C CRN, which may be described as a C-doped SiO_2_ CRN with C replacing some of the O atoms, as illustrated in Fig. 1. Its atomic structure consists of vertex-sharing tetrahedral units. Each unit has a Si atom at the center bonded to O or C atoms on the vertices. Each vertex atom is shared between two tetrahedral units and is therefore bonded to two Si atoms. Thus, there are no C-O bonds in the SiOC CRN^10^. In a C-free SiO_2_ CRN^19^, all vertex atoms are O, i.e. all tetrahedra have composition SiO_4_. By contrast, five types of tetrahedra are possible in a SiOC CRN: SiO_4_, SiO_3_C, SiO_2_C_2_, SiOC_3_, and SiC_4_^19^.

Experiments differ widely on the exact distribution of C in the SiOC CRN^4^,^5^,^10^,^19^,^20^,^25^,^26^,^27^. Some find a preponderance of SiC_4_^10^,^25^ tetrahedral units, others of SiCO_3_^4^,^19^,^20^,^27^, while some suggest there is no preference for any tetrahedral unit type^5^,^26^. There are also contradicting reports regarding the clustering tendency of C in SiOC: some propose that C aggregates^21^ while others suggest a random C distribution^19^. Several of these conflicting experiments used similar synthesis procedures and characterization techniques^19^,^25^, making it difficult to pinpoint the source of their disagreement. Our work shows that these discrepancies may be due to differing amounts of H present in the samples investigated. Consequently, our findings also suggest that it may be possible to tailor C distributions in SiOC by controlling its H content.

## Results

The clustering tendency of two dopants in a solid may be described through their interaction energy as a function of the distance between them. In crystalline solids, this quantity may be found with DFT calculations on a single series of models containing two dopants occupying successively more distant lattice positions. However, amorphous solids, such as SiOC, do not exhibit the translational invariance of perfect crystals: their atomic structure varies from location to location. Thus, the interaction energy between two C atoms in SiOC depends not only on the distance between them, but also on their location within the material. A single series of interaction energy calculations is not sufficient to characterize the clustering tendency of C. Instead, the interaction energy for two dopants at a given distance must be computed for numerous locations of the dopant pair. By repeating these calculations over a range of distances, the *average* interaction energy as a function of distance may be found.

Determining interaction energies between dopants in amorphous solids therefore poses a unique challenge for DFT calculations, which are computationally expensive: not only are a large number of calculations required, but also this number is not known *a priori*. Moreover, it is also not known how large a model must be used to sample a representative distribution of dopant locations while avoiding interactions between dopants and their periodic images. We address this challenge by a hybrid computation strategy, wherein a computationally less expensive classical potential^28^ is used to carry out a series of framing calculations, namely: to create SiO_2_ CRN models of differing sizes, determine the minimum model size that allows for accurate dopant interaction energy calculations, and estimate the number of calculations needed to compute interaction energy curves reliably. These results provide a blueprint for obtaining accurate average interaction energies with minimal computational effort using DFT calculations.

### Classical potential calculations

We used a ReaxFF^29^ classical potential for the Si-O-C system^28^ to conduct our framing simulations. This potential was developed to study oxidation of a SiC surface exposed to O_2_ and H_2_O^28^. It is therefore not transferable to SiOC. We use it only to frame subsequent DFT calculations, as described above. Amorphous CRNs of SiO_2_ are obtained by melting β-cristobalite and quenching the liquid through the glass transition down to zero temperature at rates as low as 10^11^ K/s (see Methods). All calculations are conducted under periodic boundary conditions (PBCs). Multiple properties, including density and cohesive energy of the amorphous phase, liquid phase diffusivities, and radial distribution functions (RDFs) are calculated in models containing between 192 and 1536 atoms. All behave similarly regardless of model size, demonstrating that the SiO_2_ CRN is well represented by models containing as few as 192 atoms. The RDFs we computed agree with experiments and previous simulations^30^.

Doping energies are calculated as


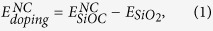


where 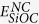 is the energy of the SiOC generated by replacing N O atoms with C in a SiO_2_ CRN and 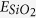 is the energy of amorphous SiO_2_ before C doping. These doping energies are to be distinguished from defect formation energies, as reference state energies for all atoms must be taken into account to obtain the latter. We do not calculate defect formation energies, since only the relative system energies are needed to obtain C-C interaction energies.

**Figure 2:**
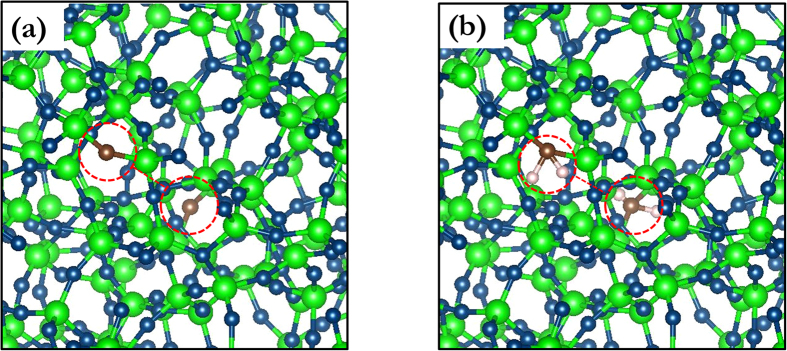
C doping in a 864-atom SiO_2_ CRN. (**a**) without H and (**b**) with H. Si atoms are large and green, O blue, C brown, and H small and pink. Positions of C atoms are shown in red circles and dashed red lines indicate C-C distances.

To study C-C interactions, two C atoms are introduced into the model simultaneously by choosing at random a pair of O atoms that falls within a pre-specified range of distances and replacing both O atoms with C, as shown in Fig. 2(a). All atomic positions in the model are then fully relaxed and the resulting doping energy, 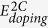, is calculated. Ranges of C-C distances are chosen so that each contains one peak of the O-O partial radial distribution function of β-cristobalite SiO_2_. They are: 0–3.6 Å, 3.6–4.7 Å, 4.7–5.4 Å, and 5.4–6.2 Å.

**Figure 3:**
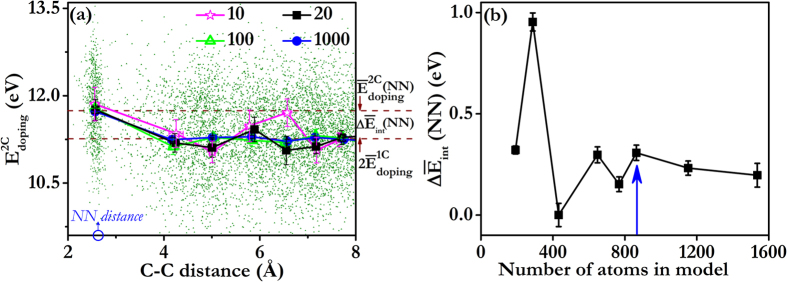
C doping and interaction energies in a SiO_2_ CRN computed using a classical potential. (**a**) C doping energies 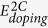 vs. C-C distance (data points). Solid lines represent averages of 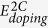 over the given number of data points. (**b**) C-C interaction energy at NN C-C distance vs. number of atoms in the model. The blue arrow indicates the model size selected or our DFT calculations.

For each range of C-C distances, we calculate 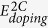 at 1000 randomly chosen locations within a model containing 864 atoms and plot the outcome in Fig. 3(a). As expected, the doping energies are always positive, consistent with the lack of stable ternary Si-C-O compounds^11^, and there is considerable scatter in 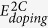 due to the lack of translational invariance in the SiO_2_ CRN. Averaging all 1000 doping energies within each distance range reveals a clear dependence of 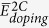 on C-C distance. Figure 3(a) shows that, on average, the C-C interaction is short ranged, as the doping energy is constant for C-C distances greater than ~4 Å. The error bars plotted in Fig. 3(a) represent standard error of the mean, computed as the standard deviation divided by the square root of the sample size.

For our DFT study, we would like to know how many doping energies must be averaged to recover the trend found using 1000 data points. Figure 3(a) shows the outcomes of averaging 10, 20, 100, and 1000 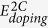 values for each range of C-C distances. The values used for these averages are selected at random from the total 1000 data points already available for each distance range. We find that the approximate dependence of 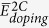 on C-C distance may be recovered using as few as 20 data points for each range of C-C distances.

To compute C-C interaction energy from doping energies, we use the expression


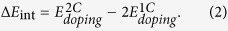


Here, 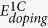 is the doping energy for one C atom. We expect interaction energies to vanish in the limit of large C-C distances. Therefore, in this limit 
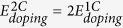
. The average doping energies in Fig. 3(a) reach this limit for C-C distances above ~4 Å, indicating that 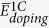 = 5.43 ± 0.04 eV. To confirm this deduction, we also calculate 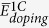 by averaging 1000 singe-C atom doping energies and find that it equals 5.28 ± 0.02 eV, close to the value reported above. Using the data in Fig. 3(a), we find the interaction energy of two C atoms at their nearest neighbor (NN) distance: 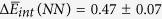 eV. This positive interaction energy signifies that, according to the Si-O-C ReaxFF classical potential we used^28^, C atoms in SiOC tend to repel, on average, and disperse uniformly throughout the material.

To calculate valid NN interaction energies under PBCs, the model must be large enough to prevent dopant pairs from interacting with their own periodic images. Simultaneously, the model should be as small as possible so that resource-intensive DFT calculations may be carried out efficiently. Figure 3(b) plots average NN interaction energies computed in models containing between 192 and 1536 atoms, demonstrating that a 864-atom model is sufficiently large for NN C-C interaction energy calculations. We further validated this conclusion by computing doping energies for C concentrations between 0% to 30% (where 100% means that all O atoms have been replaced with C). As with NN C-C interaction energies, models containing 864 atoms are sufficiently large to compute average doping energies for all C concentration.

### DFT calculations of C-C binding in SiOC

**Figure 4:**
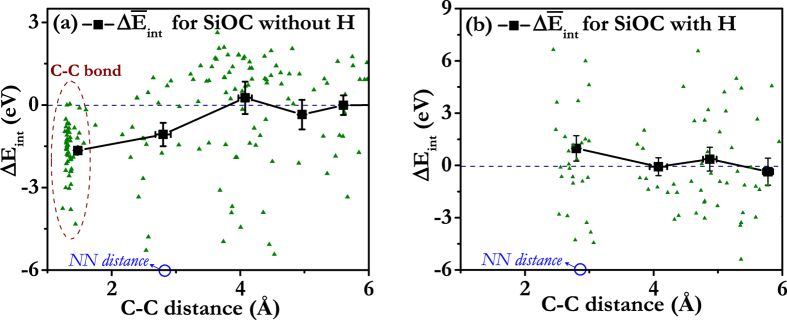
C-C interaction energy as a function of C-C distance computed using DFT. (**a**) without H and (**b**) with H. Individual data points are plotted as small triangles while averages are shown as black rectangles and solid lines. The data circled in (**a**) are NN C-C pairs whose distance decreases markedly upon relaxation.

Following the outcome of our framing study, we use DFT to determine C-C interaction energies in a 864-atom SiO_2_ CRN and average them in groups of 20 data points per C-C distance bin. C atom pairs are inserted and relaxed using the same procedure as in the classical potential calculations. Interaction energies are found by subtracting from these doping energies two times the average single atom-doping energy, 
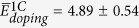
 eV (found from the asymptotic behavior of 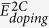 at large C-C distance, as illustrated in Fig. 3(a)). Figure 4(a) plots the resulting interaction energies—both the raw data and averages—as a function of C-C distance.

Unlike in the classical potential calculations, NN C atoms have large negative average interaction energies, i.e. they tend to bind, rather than repel. This C-C interaction is short-ranged, as the average interaction energy increases to a constant value of zero for distances greater than the NN distance. The attractive NN C-C interaction is expected to yield a tendency for C atoms to cluster. In further contrast to the classical potential calculations, some of the C-C distances change dramatically upon relaxation. In particular, the distance between some C-C pairs at NN positions decreases from ~2.6 Å to ~1.6 Å, as shown in Fig. 4(a). This reduction in NN distance does not give rise to changes in CRN topology, i.e. the tetrahedral structural units remain unbroken and joined at their vertices.

O has two-fold bonding to neighboring Si atoms in a perfect SiO_2_ CRN. Replacing O with normally four-fold bonded C leaves the latter with two unbound electrons. These electrons may be saturated with H. We therefore repeated the average interaction energy calculation described above, but added two hydrogen atoms into the interstitial sites neighboring every C substitution, as shown in Fig. 2(b). The initial C-H distance is set to the characteristic equilibrium C–H bond distance of ~1.1 Å^31^. The initial positions of the H atoms are chosen so that—together with the two NN Si atoms bonded to this C atom—they form a tetrahedral-like structure with the C atom in the middle. The average single atom-doping energy with H passivation is 
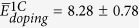
 eV.

Figure 4(b) shows the average interaction energy as a function of average C-C distance for H-passivated C. Due to the greater structural variability arising from the added H atoms, the individual interaction energies are scattered over a larger energy range than in Fig. 4(a) (over ~15 eV, as compared to ~9 eV without H). The uncertainties of the average C-C interaction energies with H are therefore larger than without H. Nevertheless, the effect of H may be clearly seen: C atoms at NN locations have positive interaction energy, i.e. they do not bind. Instead, they prefer to be separated to distances greater than the NN C-C distance. This effective repulsion leads to uniform dispersal of C throughout the CRN and enrichment of SiCO_3_ tetrahedral units. Moreover, there is no tendency for the NN C-C distance to undergo a marked reduction upon relaxation, in contrast with the case of C doping without H.

## Discussion

We have shown that H has a substantial effect on the average clustering tendency of C atoms in SiOC: without H, C tends to cluster; with H, it tends to disperse uniformly. Some experiments on SiOC indicate C aggregation and enrichment of SiC_4_ structural units^10^,^21^,^25^ while others claim that C is uniformly distributed, enhancing the number of SiCO_3_ units^4^,^5^,^19^,^20^,^26^,^27^. Our finding suggests that these discrepancies may be due to differing amounts of H present in the samples investigated in these studies. Indeed, SiOC is unlikely to ever be completely H-free: H is commonly found in SiOC precursors used in chemical vapor deposition (CVD)^5^ and sol-gel methods^4^. A certain amount of it is also retained after pyrolysis^4^,^32^,^33^,^34^. Our work furthermore suggests that changing H content may provide a means to tuning the C distribution in SiOC.

Often, the amount of retained H is not measured in experiments, making it difficult to correlate reported C clustering behavior to H content. However, our explanation is consistent with the work of Liang *et al.*, who observed a decrease in the number of SiC_4_ and SiC_2_O_2_ tetrahedral units and an increase in the number of SiCO_3_ units when SiOC pyrolysis is performed in the presence of water vapor, which may serve as a H source^35^. Some have further proposed that H may contribute to increased thermodynamic stability of SiOC^34^.

**Figure 5:**
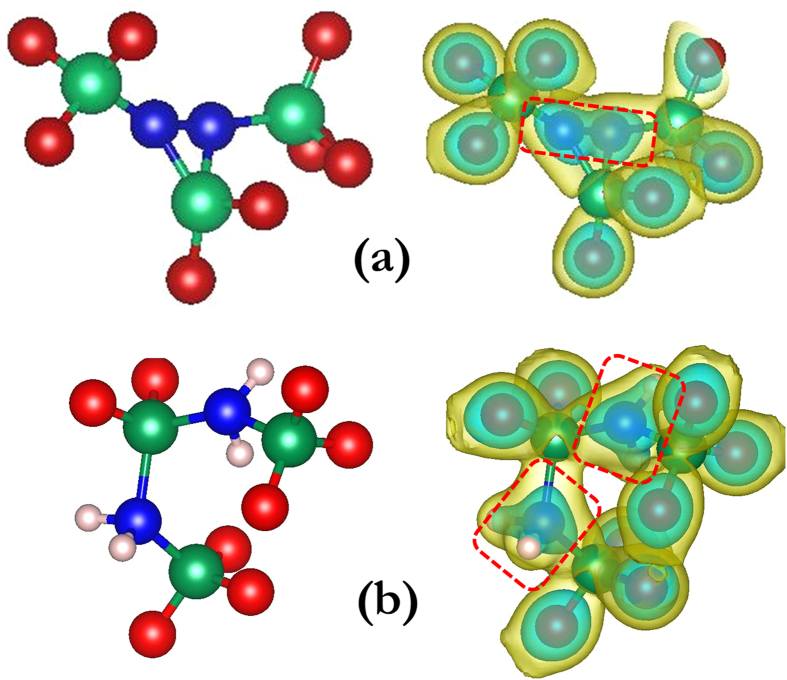
Charge density plots showing bond configurations near neighboring C atoms. (**a**) without H and (**b**) with H. The left column shows atoms alone while the right one additionally shows electron density isosurfaces (outer yellow: 0.05 *e*/Å^3^; inner cyan: 0.15 *e*/Å^3^). The C-C bond in (**a**) and C–H bonds in (**b**) are indicated with dashed red rectangles. C is blue, O red, Si large and green, and H small and pink.

The attractive interaction between C atoms in H-free SiOC is primarily due to the formation of direct C-C bonds when two C atoms appear in the same tetrahedral unit or are otherwise close to each other. This interpretation is consistent with the reduction of the C-C distance to ~1.6 Å—comparable to the typical C-C bond length of ~1.5 Å^31^—upon relaxation of the C-doped SiO_2_ CRN. To further validate this interpretation, we plot electronic charge densities around C atoms in Fig. 5. The two C atoms in the H-free model in Fig. 5(a) are contained within the same electron density isosurface (0.15 *e*/Å^3^), confirming the presence of a direct bond between them. Such C-C bonds may initiate the formation of a phase-separated carbon network in SiOC^19^,^23^,^24^.

Hydrogen saturates the C bonding environment, preventing the formation of direct C-C bonds. The charge density isosurfaces in Fig. 5(b) show direct bonding of H to C and no direct C-C bond. The repulsive C-C interaction seen upon introduction of H may arise from misfit strains, wherein a H atom occupying an interstitial site distorts neighboring interstitials sites, inhibiting other H atoms from occupying them. To further motivate this interpretation, we computed H-C-H, Si-C-Si, and Si-C-H angles for different C-C distances. For NN C atom pairs the H-C-H, Si-C-Si, and Si-C-H angles are ~90.6 ± 2.8°, ~133.8 ± 2.4°, and ~104.6 ± 2.2°, while for larger distances the angles are closer to ~83.3 ± 1.9°, ~141.8 ± 1.4°, and ~105.4 ± 1.6°, respectively. Thus, the proximity of H-terminated C atoms gives rise to marked distortions in the C bonding environment.

In the absence of H, some NN C pairs do not form direct C-C bonds, but nevertheless have negative interaction energies, as may be seen in Fig. 4(a). One possible reason is the electronegativity difference between C and O, which may also contribute to effective attractive interactions between C atoms in H-free SiOC. The electronegativity difference between C and O modifies the effective charge of Si atoms at the centers of tetrahedral units, depending on the C and O occupancy of tetrahedral unit vertices. When a C atom bonds to a Si atom by replacing one of its O neighbors, the effective charge on the Si decreases, thereby weakening the other Si-O bonds. The remaining O atoms are therefore easier to replace, favoring the formation of C-rich tetrahedral units. The decreased effective charge on Si is also expected to cause the bond lengths of the remaining Si-O bonds in a C-containing tetrahedral unit to increase. Indeed, our DFT calculations show a ~0.1 Å increase in Si-O bond lengths in such tetrahedra.

Finally, the present study demonstrates a hybrid classical potential/first principles approach to investigating the properties of amorphous solids. The former is used to perform computationally inexpensive preliminary scoping calculations that frame subsequent computationally demanding, high-accuracy first principles calculations. The DFT calculations performed here may also be used to further refine existing classical potentials^29^, extending their transferability to SiOC.

## Methods

Classical potential calculations were performed with LAMMPS^36^ using a 0.5 femtosecond time step. Crystalline models were melted by heating to 5000 K in 25 K increments with a 4000 MD step equilibration after each increment. Quenching was conducted using 10 K temperature decrements, each followed by 20000 or 200000 MD steps, corresponding to quench rates of 10^12^ K/s and 10^11^ K/s, respectively. A Nosé–Hoover^37^,^38^ thermostat and barostat was used to maintain constant zero pressure. The amorphous structures obtained using both quench rates were comparable, though slower quenching yielded a lower final density.

DFT calculations were performed in supercells containing 864 atoms using VASP^39^, a plane wave based first-principles DFT code. We used the Perdew-Burke-Ernzerhof (PBE)^40^ exchange-correlation functional within projector-augmented-wave approach^41^, a gamma-point only k point mesh, a 300 eV plane wave kinetic energy cutoff, and an energy convergence threshold of 10^−4^ eV for the electronic self-consistent loop. Soft pseudopotentials of oxygen and carbon, as well as standard pseudopotential for Si were used (O_s, C_s, and Si in VASP’s nomenclature, respectively). An energy convergence criterion of 10^−3^ eV was used for ionic relaxations within the conjugate gradient minimization scheme.

## Additional Information

**How to cite this article**: Ding, H. and Demkowicz, M. J. Hydrogen reverses the clustering tendency of carbon in amorphous silicon oxycarbide. *Sci. Rep.* **5**, 13051; doi: 10.1038/srep13051 (2015).

## Figures and Tables

**Figure 1:**
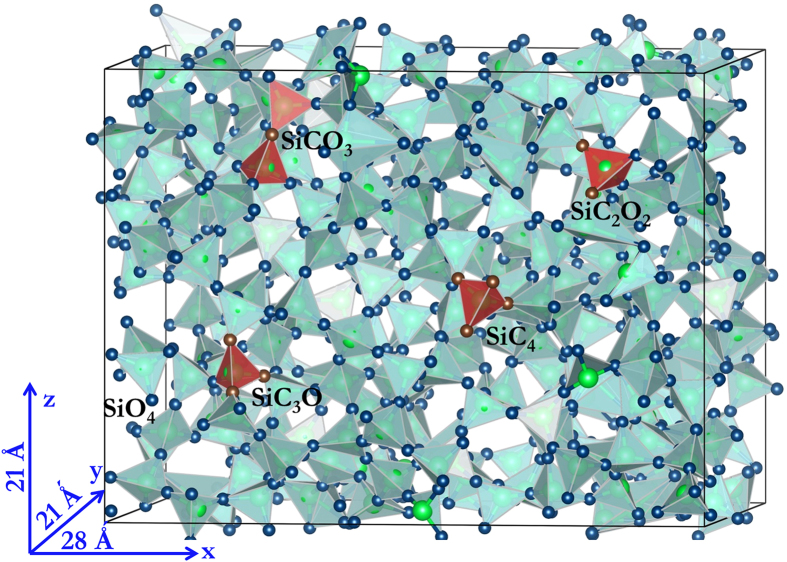
Continuous random network (CRN) of SiOC. Si atoms are large green, O blue, and C brown. Examples of the five distinct types of vertex-sharing tetrahedral units found in SiOC are indicated.
